# Limited additional value of dual-layer spectral 4DCT compared with conventional 4DCT for preoperative localization in primary hyperparathyroidism

**DOI:** 10.1016/j.ejro.2025.100669

**Published:** 2025-06-24

**Authors:** Jorian P. Krol, Tessa Veerbeek, Laura N. Deden, Frank B.M. Joosten, Marie Louise E. Bernsen, Cornelis H. Slump, Wim J.G. Oyen

**Affiliations:** a Department of Radiology & Nuclear Medicine, Rijnstate Hospital, Arnhem, the Netherlands; b Department of Robotics and Mechatronics, Faculty of Electrical Engineering, Mathematics and Computer Sciences, University of Twente, Enschede, the Netherlands; c Humanitas University, Department of Biomedical Sciences and Humanitas Clinical and Research Centre, Department of Nuclear Medicine, Milan, Italy; d Radboud University Medical Centre, Department of Radiology & Nuclear Medicine, Nijmegen, the Netherlands

**Keywords:** Primary hyperparathyroidism, Parathyroid adenoma, 4DCT, Dual-layer CT, Spectral CT

## Abstract

**Purpose:**

Primary hyperparathyroidism, characterized by excessive parathyroid hormone secretion, is typically caused by solitary parathyroid adenomas or multiglandular disease. Accurate preoperative localization is critical for successful surgical parathyroidectomy. While four-dimensional CT (4DCT) is commonly used for this purpose, spectral-CT techniques have recently been introduced, offering improved tissue differentiation. Rapid kV switching and dual-source spectral-CT have been studied, however, this is the first study that evaluates the effectiveness of dual-layer-CT in preoperatively locating parathyroid adenomas in a larger population.

**Approach:**

From April 2020 to October 2023, patients with confirmed primary hyperparathyroidism underwent dual-layer spectral 4DCT before surgery. Spectral reconstructions (MonoE40keV, Iodine-Density, Z-effective, Iodine-no-Water, Virtual Non-Contrast) were analyzed alongside conventional CT reconstructions. Mean attenuation values were compared using one-way ANOVA. ROC curves with paired-sample analysis assessed the ability of different reconstructions to distinguish between thyroid and parathyroid tissue, and lymph nodes and parathyroid tissue.

**Results:**

Thirty-six patients with thirty-nine parathyroid adenomas were analyzed. Conventional CT reconstructions demonstrated significantly higher AUC values for differentiating thyroid from parathyroid tissue across all phases compared to spectral reconstructions (0.83–0.95 vs. 0.65–0.89, p-value 0.007-<0.001). No significant difference was found between conventional and spectral reconstructions in distinguishing lymph nodes from parathyroid tissue (0.64–0.96 vs. 0.58–0.96, p-value 0.070–0.957). Virtual non-contrast (VNC) reconstructions showed smaller attenuation differences and lower AUC values in arterial and delayed phases compared to true non-contrast (p = 0.031 and 0.034).

**Conclusions:**

Dual-layer spectral-CT is comparable or inferior to conventional CT in tissue differentiation. VNC reconstructions are not recommended as a substitute for true non-contrast due to inconsistent results. In this cohort, dual-layer spectral 4DCT did not demonstrate clear clinical advantage; further validation is warranted.

## 1 Introduction

Primary hyperparathyroidism (pHPT) is an endocrine disorder caused by excess parathyroid hormone (PTH) secretion by single or multiple parathyroid glands [1], [2]. The estimated prevalence of pHPT is one to seven cases per thousand adults [3]. Typically, individuals have four parathyroid glands (84 %), located along the thyroid lobes [4]. Abnormal embryonal migration can result in ectopic glands which can be located anywhere from the mandible angle to the mediastinum and exist in 16 % of patients with pHPT [5], [6]. pHPT is caused by a solitary parathyroid adenoma (PA) in 80–85 % of the cases. Multiglandular disease accounts for 15 %-20 % of the cases [7], [8]. Treatment of pHPT consists of surgical excision of the hyperfunctioning PA. The preferred surgical option is minimally invasive parathyroidectomy, as it results in a shorter duration of hospital admission, fewer complications, and smaller incisions compared to conventional bilateral neck exploration. Accurate preoperative localization of the PA is crucial to allow successful minimally invasive parathyroidectomy.

Currently, four-dimensional computed tomography (4DCT) is increasingly used for preoperative localization of the PA. It generally consists of a non-enhanced phase followed by an intravenous injection of an iodine-based contrast agent, an arterial phase and a venous phase or a delayed phase or a combination thereof. It exploits perfusion characteristics to identify the highly vascularized PA from mimics such as the thyroid gland and lymph nodes [9]. Three different types of enhancement patterns of PAs have been reported, which makes accurate localization even more difficult [10]. Furthermore, scan protocols vary largely in number of phases and phase timing among hospitals, resulting in widespread sensitivity and specificity [11].

Recently, spectral CT techniques (e.g. dual-source, rapid kV switching, and dual-layer) have become clinically available and allow for extended tissue differentiation [12]. Distinguishing PAs from their mimics was more successful using low energy reconstructions from spectral CT than conventional reconstructions [13], [14], [15], [16], [17]. Moreover, the use of spectral reconstructions that visualize atomic numbers (Z-effective reconstruction), or iodine concentration (Iodine Density/Iodine no Water) are potentially useful as well [13], [14], [15], [16].

Besides, from spectral CT virtual non-contrast (VNC) reconstructions can be created. These could theoretically replace the true non-contrast or true non-enhanced (TNC) phase by filtering out the iodine content and thereby leading to a reduction in radiation dose. Current studies are inconsistent in the use of a VNC scan. Some studies report that VNC scans can be used as an alternative to TNC, although most studies conclude that VNC reconstructions cannot be used because of the intrinsic iodine content of the thyroid [18], [19].

These previous results were mainly based on dual-source or rapid kV techniques, only one case report by Maraia et al. described the use of dual-layer spectral CT [19]. The potential benefit of dual-layer technique compared to the dual-source and rapid kV switching is the excellent spatial and temporal resolution, due to the simultaneous aligned acquisition [20]. Therefore, in the current study, we investigate the benefit of dual-layer spectral 4DCT for the improvement of preoperative localization of PAs in pHPT patients. We hypothesized that dual-layer spectral CT would provide extra information as compared to conventional 4DCT and that it could improve the differentiation of PAs from thyroid tissue. Also, the use of VNC as a replacement for TNC was investigated.

## 2 Materials and methods

### 2.1 Patient population

Patients with biochemically proven pHPT who underwent spectral 4DCT between April 2020 and October 2023 for preoperative localization before undergoing parathyroidectomy, were retrospectively included in this study. Surgical and histopathological confirmation of single- or double-gland disease as an inclusion criterium. Preoperative pHPT diagnosis was based on elevated serum level of PTH > 9.3 pmol/L and calcium > 2.60 mmol/L. Patients who had not undergone surgery or had negative surgery were excluded. Patients with previous neck surgery were also excluded. This study was approved by the institutional research ethics board (Research number 2022–2154), which waived the requirement of written informed consent of each individual patient.

### 2.2 Scan protocol

The 4DCT scans were acquired on the iQon dual-layer spectral CT scanner (Philips Medical Systems, Best, the Netherlands) at the Rijnstate Hospital (Arnhem, The Netherlands) following a standardized protocol. Patients were placed in a supine position with their arms alongside their body. The scanning protocol consisted of four identical helical scans obtained in an automated, predetermined, and timed sequence. After the non-contrast scan was performed, iodinated contrast medium (Optiray 300 mg I/mL, Guerbet, Villepinte, France) was administered intravenously followed by a 30 mL saline flush. The volume and flow of the administered contrast depended on the patient’s weight and varied between 90 mL – 120 mL and 3.5 mL/s – 5.0 mL/s, respectively. Bolus tracking in the ascending aorta was applied for phase timing. The arterial, venous, and delayed phases were scanned at 10, 40, and 85 s after the bolus tracking threshold of 150 HU, respectively. The average dose length product was 497 mGy x cm, with a calculated effective dose of 5.6 mSv.

### 2.3 Reconstructions

Conventional CT reconstructions were reconstructed using the regular hybrid iterative reconstruction (iDose) protocol (slice thickness 0.90 mm, pixel spacing 0.5 mm, tube current 234 mA, x-ray tube voltage 120 kVp). Spectral reconstructions were generated using Phillips IntelliSpace Portal (v11, Philips Healthcare USA) with a similar thickness as conventional CT. Spectral reconstructions were selected based on previous studies and user experience: 1) MonoE40keV, 2) Iodine Density, 3) Z-effective, 4) Iodine no Water, and 5) Virtual Non-Contrast (VNC). The first four reconstructions were intended to improve tissue differentiation (in particular between PA and thyroid). The VNC reconstruction was analyzed as an alternative to the TNC scan. Spectral reconstructions were made from all available phases to gather as much information as possible. The VNC reconstruction from the TNC was made to use as a control, as theoretically only the thyroid iodine should be extracted from the TNC to generate the VNC.

### 2.4 Analysis

Region of interests (ROIs) for the PA, thyroid, and lymph nodes were drawn, preferably in a single slice to minimize the effect of artefacts or surrounding tissue. Location of the PA ROI was correlated with the surgical location. ROIs were drawn by J.K. and checked by T.V. for any inclusion of surrounding tissue. Then, J.K. and T.V. checked all final ROIs again and the raw data to assure quality of the ROIs. If the lymph node was too small (<5 mm short axis) to draw an ROI, a lymph node in level I or II with a short axis of at least 5 mm was used and the fatty hilum was not included in the ROI. For all three tissue types, the mean attenuation was calculated in all spectral reconstructions for each individual scan. For reconstructions that were used to improve tissue differentiation, the mean attenuation was visualized in a contrast enhancement graph. Furthermore, ROC analysis was performed for conventional CT and all reconstructions to determine the ability to differentiate between thyroid and parathyroid and between lymph node and parathyroid. For the VNC reconstruction, the mean attenuations were compared to the TNC attenuation.

For comparison, patients were categorized by the enhancement pattern of the parathyroid gland in relation to the thyroid gland as mentioned by Bahl et al [10]. A type A enhancement pattern of a PA is lower attenuation on the non-enhanced phase, higher attenuation on the arterial phase, and lower attenuation on the venous and delayed phase, in relation to the thyroid gland. A type B enhancement pattern is lower attenuation of the PA compared to the thyroid in all phases. Type C enhancement pattern is lower attenuation on the non-enhanced and arterial phase but higher attenuation on the venous or delayed phase.

### 2.5 Statistics

One-way ANOVA was used to determine statistically significant differences between the different tissue types within each reconstruction, within each phase.

ROC curves comparing each reconstructions’ ability to differentiate between thyroid and parathyroid and between lymph node and parathyroid were plotted with a paired-sample analysis. For example, in conventional and MonoE40keV reconstructions, sensitivity and specificity for every HU value for differentiation between two tissue types were calculated, creating an ROC curve. This was repeated for each reconstruction and was performed to compare the different reconstructions with different units. A p-value of 0.05 or less was considered significant. Data was split for each phase.

One-way ANOVA was used to determine statistically significant differences between the different tissue types for each VNC reconstruction compared to the TNC. Posthoc analysis, Bonferroni or, if homogeneity of variance was doubtful, Games-Howell, was applied to determine between which groups a significant difference exists. ROC analysis with paired-sample analysis was performed to compare AUC values of each VNC reconstruction to the TNC.

## 3 Results

### 3.1 Patient population

Thirty-six patients (27 females, mean age 61, range 26 – 79) with single (91.7 %) or double (8.3 %) gland pHPT met all criteria and were included. The clinical and biochemical characteristics of patients are summarized in Table 1. All PAs were surgically proven, and the surgical location was correlated with the ROI location of the PA. Thirty-five out of 39 parathyroid adenomas (89.7 %) had lower attenuation than the thyroid gland in all phases, classified as type B enhancement. The other four parathyroid adenomas (10.2 %) had lower attenuation on the non-enhanced phase, higher attenuation on the arterial phase, and lower attenuation on the venous and delayed phase, in relation to the thyroid gland (type A enhancement pattern). Because of the small number of type A enhancement pattern, no subgroups were analyzed.

**Table1:** Patient characteristics.

Characteristics	Total (n = 36)	Type A enhancement pattern (n = 4)	Type B enhancement pattern (n = 32)
Age (y), mean ±SD (range)	61 ± 12 (26 – 79)	60 ± 4 (54 – 63)	62 ± 12 (26−79)
Female: Male ratio	27:9	2:2	25:7
Serum calcium mmol/L (range)	2.83 (2.35–3.45)	2.67 (2.61 – 2.73)	2.80 (2.35–3.45)
Serum PTH pmol/L (range)	23.9 (5.7 – 111.3)	28.2 (10.6 – 53.2)	23.4 (5.7 – 111.3)
Maximum tumor diameter (CT), mm (range)	12 (5 – 30)	13 (5 – 20)	12 (5 – 30)
Maximum tumor diameter (histopathology), mm (range)	20 (8 – 43)	25 (15 – 35)	19 (8 – 43)
Maximum tumor weight (histopathology), g (range)	1.4 (0.1 – 8.2)	2.9 (0.3 – 8.2)	1.2 (0.1 – 6.5)

### 3.2 ANOVA and ROC analysis spectral reconstructions

**Fig. 1:**
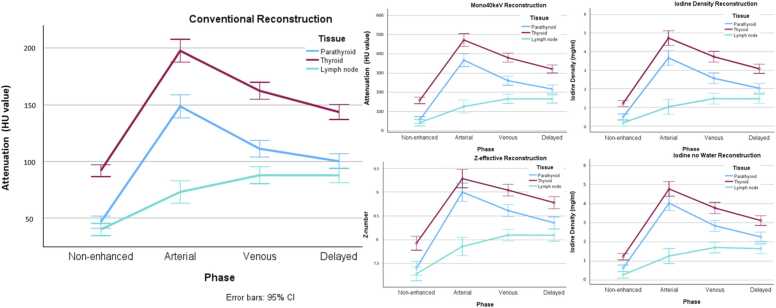
Mean attenuation graph of the population for conventional (1), Mono40keV (2), Iodine Density (3), Z-effective (4) and Iodine No Water (5) reconstruction. These graphs show the attenuation (1 & 2), iodine density (3 & 5) or effective Z-number of the thyroid, PA or lymph node in the four different phases. It shows a lower attenuation for the PA compared to the thyroid in all phases and shows nearly identical graphs for the spectral reconstructions. Lymph node attenuation was the lowest in all phases.

**Fig. 2:**
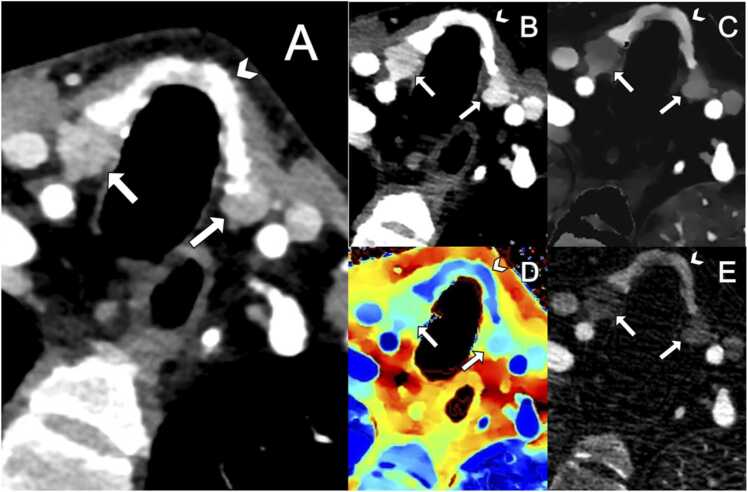
An example of bilateral type B enhancement PA in arterial phase in Conventional (A), Mono40keV (B), Iodine Density (C), Z-effective (D) and Iodine no Water (E) reconstruction. Arrowhead shows the thyroid and the arrows show the type B enhancement PAs.

Mean attenuation of each tissue type for each phase and reconstruction is shown in Table 2. The attenuation graphs of each tissue type for each phase and reconstruction are shown in Fig. 1. An example of a PA in the arterial phase in each reconstruction is shown in Fig. 2. There was a significant difference in attenuation between the PA and thyroid in each phase, except for the Z-effective and Iodine no Water reconstructions in the arterial phase. There was a significant difference in attenuation between the PA and lymph nodes in each phase, except for the Conventional and Mono40keV reconstruction of the non-enhanced phase and VNC reconstruction of venous and delayed phase.

**Table 2:** Values of each tissue type in every phase and reconstruction with 95 % interval.

Reconstruction	Tissue	Non-enhanced mean (95 % CI)	Arterial mean (95 % CI)	Venous mean (95 % CI)	Delayed mean (95 % CI)
Conventional (HU value)	Parathyroid	**46.3** (40.7–51.9)	**148.7** (136.7–160.6)	**111.1** (102.6–119.6)	**100.1** (92.8–107.4)
	Thyroid	**91.8** (84.5–99.1)	**197.7** (185.8–209.6)	**162.3** (153.3–171.3)	**143.6** (135.7–151.6)
	Lymph node	**39.8** (37.5–42.1)	**73.0** (67.3–78.6)	**87.8** (83.2–92.3)	**87.7** (83.0–92.3)
Mono40keV (HU value)	Parathyroid	**56.8** (38.1–75.6)	**366.7** (326.8–406.6)	**260.4**(234.5–286.4)	**216.8** (192.6–240.9)
	Thyroid	**157.5** (135.0–180.0)	**471.0** (430.0–512.0)	**379.7** (348.9–410.4)	**320.9** (294.3–347.5)
	Lymph node	**42.5** (37.9–47.2)	**126.5** (113.3–139.7)	**165.9** (154.0–177.7)	**165.6** (155.3–176.0)
Iodine Density (mg/mL)	Parathyroid	**0.47** (0.30–0.65)	**3.66** (3.16–4.17)	**2.56** (2.25–2.88)	**2.03** (1.73–2.32)
	Thyroid	**1.20** (0.97–1.43)	**4.73** (4.27–5.19)	**3.72** (3.35–4.10)	**3.08** (2.78–3.38)
	Lymph node	**0.16** (0.11–0.22)	**1.03** (0.88–1.17)	**1.47** (1.32–1.61)	**1.46** (1.34–1.58)
Z-Effective (Z-number)	Parathyroid	**7.40** (7.22–7.59)	**9.00** (8.76–9.24)	**8.61** (8.47–8.75)	**8.36** (8.21–8.50)
	Thyroid	**7.93** (7.76–8.09)	**9.28** (9.06–9.50)	**9.04** (8.89–9.19)	**8.78** (8.62–8.93)
	Lymph node	**7.29** (7.22–7.36)	**7.86** (7.78–7.96)	**8.10** (8.03–8.17)	**8.10** (8.04–8.15)
Iodine no Water (mg/mL)	Parathyroid	**0.63** (0.45–0.81)	**4.02** (3.54–4.51	**2.84** (2.52–3.16)	**2.26** (1.97–2.55)
	Thyroid	**1.23** (0.99–1.46)	**4.77** (4.30–5.24)	**3.78** (3.40–4.15)	**3.12** (2.82–3.42)
	Lymph node	**0.28** (0.24–0.32)	**1.26** (1.11–1.41)	**1.71** (1.57–1.84)	**1.64** (1.50–1.78)
Virtual Non-Contrast (HU value)	Parathyroid	**32.2** (27.5–36.9)	**49.4** (43.4–55.5)	**41.5** (36.2–46.8)	**44.4** (39.7–49.1)
	Thyroid	**58.7** (55.9–61.4)	**69.9** (66.5–73.3)	**62.4** (59.4–65.3)	**60.2** (57.3–63.2)
	Lymph node	**32.9** (29.7–36.0)	**36.5** (33.2–39.8)	**40.1** (37.5–42.6)	**40.3** (37.6–43.0)

**Fig. 3:**
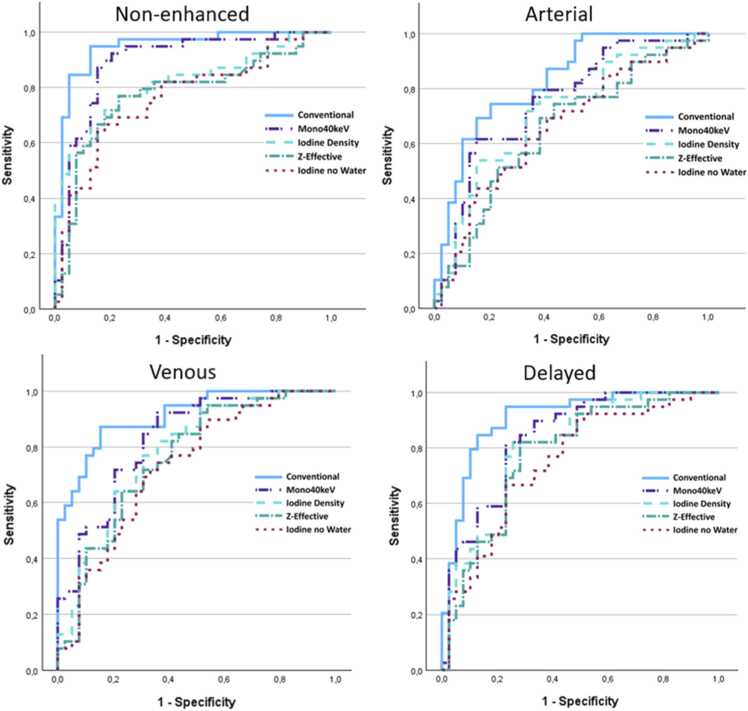
ROC curves for tissue differentiation between thyroid and PA for conventional and spectral reconstructions. Graphs are shown for each phase.

ROC analysis for each reconstruction in differentiation between thyroid and parathyroid showed AUC values for each reconstruction in each phase between 0.651 and 0.949 (see Table 3). The conventional CT reconstruction showed significantly higher AUC values for each phase compared to each spectral reconstruction (AUC difference between 0.057 and 0.183, p-value 0.007-<0.001, see Table 4). ROC curves for each phase are shown in Fig. 3.

**Table 3:** AUC for each reconstruction in differentiating between thyroid and parathyroid.

Reconstruction	Non-enhanced (95 % CI)	Arterial (95 % CI)	Venous (95 % CI)	Delayed (95 % CI)
Conventional	**0.949** (0.900–0.997)	**0.829** (0.739 – 0.919)	**0.913** (0.852 – 0.973)	**0.909** (0.842 – 0.976)
Mono40keV	**0.892** (0.815 – 0.969)	**0.753** (0.643 – 0.862)	**0.828** (0.738 – 0.919)	**0.840** (0.752 – 0.929)
Iodine Density	**0.819** (0.724 – 0.915)	**0.714** (0.598 – 0.830)	**0.781** (0.679 – 0.884)	**0.804** (0.706 – 0.902)
Z-Effective	**0.777** (0.668 – 0.887)	**0.651** (0.527 – 0.775)	**0.758** (0.651 – 0.866)	**0.781** (0.677 – 0.885)
Iodine no Water	**0.766** (0.658 – 0.874)	**0.661** (0.538 – 0.783)	**0.730** (0.618 – 0.843)	**0.751** (0.642 – 0.860)

**Table 4:** Difference in AUC between conventional CT and different spectral reconstructions in differentiating between thyroid and parathyroid.

Reconstruction	Non-enhanced AUC difference (p-value)	Arterial AUC difference (p-value)	Venous AUC difference (p-value)	Delayed AUC difference (p-value)
Conventional – Mono40keV	**0.057** (0.007)	**0.076** (0.005)	**0.084** (0.003)	**0.068** (0.007)
Conventional – Iodine Density	**0.130** (0.002)	**0.115** (0.001)	**0.131** (<0.001)	**0.105** (0.003)
Conventional – Z-Effective	**0.171** (<0.001)	**0.178** (<0.001)	**0.154** (<0.001)	**0.128** (0.001)
Conventional – Iodine no Water	**0.183** (<0.001)	**0.168** (<0.001)	**0.182** (<0.001)	**0.158** (<0.001)

**Fig. 4:**
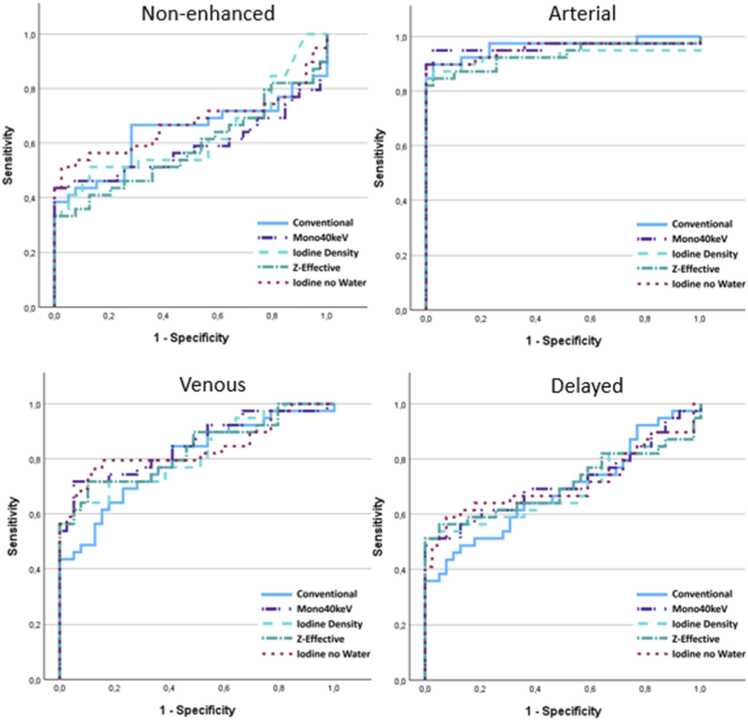
ROC curves for tissue differentiation between lymph node and PA for conventional and spectral reconstructions. Graphs are shown for each phase.

ROC analysis for each reconstruction in differentiation between lymph nodes and parathyroid showed AUC values for each reconstruction in each phase between 0.584 and 0.964 (see Table 5). There was no significant difference in AUC values for each phase for conventional CT reconstruction compared to spectral reconstructions (AUC difference −0.053–0.057, p-value 0.091–0.957, see Table 6). ROC curves for each phase are shown in Fig. 4.

**Table 5:** AUC for each reconstruction in differentiating between lymph node and parathyroid.

Reconstruction	Non-enhanced (95 % CI)	Arterial (95 % CI)	Venous (95 % CI)	Delayed (95 % CI)
Conventional	**0.641** (0.508 – 0.774)	**0.964** (0.921 – 1.007)	**0.798** (0.700 – 0.897)	**0.687** (0.567 – 0.807)
Mono40keV	**0.588** (0.449 – 0.727)	**0.962** (0.907 – 1.016)	**0.851** (0.764 – 0.938)	**0.716** (0.596 – 0.836)
Iodine Density	**0.626** (0.495 – 0.757)	**0.927** (0.853 – 1.001)	**0.820** (0.726 – 0.914)	**0.690** (0.563 – 0.816)
Z-Effective	**0.584** (0.452 – 0.717)	**0.930** (0.865 – 0.995)	**0.831** (0.739 – 0.924)	**0.707** (0.582 – 0.831)
Iodine no Water	**0.671** (0.540 – 0.803)	**0.954** (0.899 – 1.009)	**0.836** (0.740 – 0.932)	**0.711** (0.587 – 0.835)

**Table 6:** Difference in AUC between conventional CT and different spectral reconstructions in differentiating between lymph node and parathyroid.

Reconstruction	Non-enhanced AUC difference (p-value)	Arterial AUC difference (p-value)	Venous AUC difference (p-value)	Delayed AUC difference (p-value)
Conventional – Mono40keV	**0.053** (0.309)	**0.002** (0.856)	**−0.053** (0.091)	**−0.029** (0.441)
Conventional – Iodine Density	**0.015** (0.840)	**0.037** (0.170)	**−0.022** (0.610)	**−0.003** (0.957)
Conventional – Z-Effective	**0.057** (0.430)	**0.034** (0.070)	**−0.033** (0.453)	**−0.020** (0.699)
Conventional – Iodine no Water	**−0.030** (0.671)	**0.010** (0.303)	**−0.038** (0.423)	**−0.024** (0.601)

### 3.3 Virtual non-contrast reconstruction

**Fig. 5:**
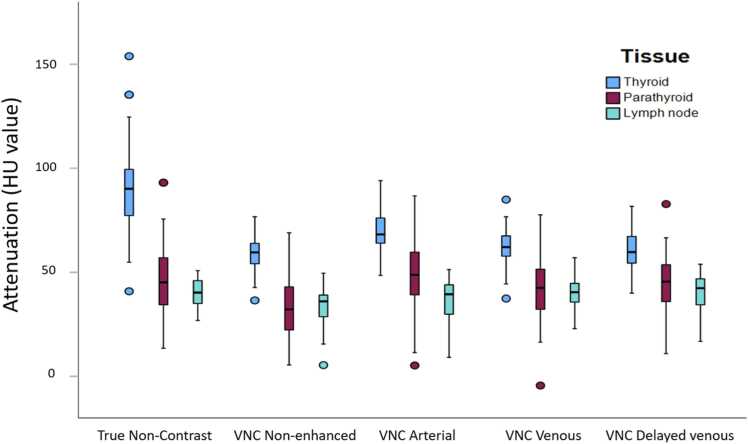
This figure shows the mean attenuation for the three tissue types (Thyroid, PA and lymph node) on the TNC compared to VNC reconstructions from non-enhanced, arterial, venous and delayed venous phase.

VNC reconstructions were generated for all four phases. The VNC of the true non-enhanced (TNC) phase was created for theoretical purpose to see if only the attenuation of the thyroid gland would have changed, however VNC reconstruction of the non-enhanced phase was not used in the ANOVA and ROC analysis. The mean attenuation of all three tissue types was compared using the TNC phase as a reference, see Fig. 5. In all VNC reconstructions, attenuation of the thyroid decreased compared to the TNC (60.2–69.9 HU compared to 91.8 HU, see Table 2). The attenuation of PAs and lymph nodes remained similar, though varied among VNCs in comparison to TNC. Attenuation of PAs varied between 41.5 and 49.4 HU compared to 46.3 HU in the TNC. Attenuation of lymph nodes varied between 36.5 and 40.3 HU compared to 39.8 HU in the TNC.

There is a significant difference in attenuation between thyroid and PA in the TNC and each VNC reconstruction (p-value <0.001), however the difference between thyroid and PA varied between 15.8 and 20.9 HU compared to 45.5 HU in the TNC.

ROC analysis showed an AUC of 0.95 for the non-enhanced phase to differentiate between thyroid and PA. The AUC of the VNC reconstructions of the arterial, venous and delayed phase were 0.83, 0.88 and 0.84 respectively. There was a significant difference in AUC between the non-enhanced phase and the VNC reconstruction from the arterial and delayed phase (p-value of 0.031 and 0.034 respectively).

## 4 Discussion

In this study, the potential benefits of dual-layer spectral 4DCT to improve preoperative localization of PAs in pHPT patients were analyzed. In this cohort, there was a significant attenuation difference between the three tissue types in most spectral reconstructions. However, the spectral reconstructions showed a significant lower AUC compared to the conventional reconstruction for differentiating between thyroid and PA and showed similar AUC for differentiating between lymph nodes and PA. The spectral reconstructions furthermore showed a wider 95 % confidence interval compared to the conventional reconstruction. Also, the utility of VNC as a replacement for TNC is limited, as the VNC seemed to be inconsistent and showed a smaller difference in attenuation between the thyroid and PA compared to the TNC. Our study confirmed the high incidence of type B enhancement in PA [10]. Furthermore, our data also showed a small difference in results between the different enhancement types, namely that spectral reconstructions could show higher tissue differentiation between thyroid and PA, especially in the arterial phase and Mono40keV reconstruction for type A enhancement. However, no proper statistical test could be performed because of the small amount of type A enhancement types.

A previous study by Woisetschläger et al. showed significantly different attenuation for PAs, thyroid, and lymph nodes in different phases, with a larger difference in lower keV values using spectral CT data [16]. Bunch et al. showed significantly increased attenuation for the thyroid gland compared to the parathyroid lesion in 40 keV virtual monoenergetic images compared to 70 keV [17]. Forghani et al. used a dual-energy CT with only two phases to compare HU attenuation characteristics of PAs, thyroid, and lymph nodes. Attenuation was significantly different between PA and lymph nodes in the 25-second phase and between PA and thyroid in the 55-second phase in lower keV values [13].

The use of VNC as a replacement for TNC is limited according to our study, as the VNC seems to be inconsistent. The thyroid gland contains native iodine which was used as a feature to distinguish the PA from the thyroid gland. Furthermore, due to the intrinsic iodine content of the thyroid, a lower mean attenuation was expected for the thyroid on all reconstructed VNCs compared to the TNC. The thyroid demonstrated a higher attenuation compared to the PA and lymph node in all VNCs. However, the difference in attenuation was so small that its clinical relevance is uncertain. In all VNC reconstructions, the mean attenuation of PAs and lymph nodes was not as expected. There was a big variation in the attenuation of both PAs and lymph nodes. In VNCs of type A PAs, it seemed that the enhancement pattern over time was recognizable in the VNC reconstructions for all tissue types. The inconsistent iodine removal of VNCs was supported by the non-enhanced VNC: while there was no iodine present in the PA or lymph node, the VNC reconstruction showed a lower attenuation for both tissues when compared to the conventional TNC.

This is in line with most literature. Roskies et al., Forghani et al., and Woisetschläger et al. all showed the VNC to be not useful because of the decreased tissue differentiation between PA and thyroid [13], [14], [16], [21]. Leiva-Salinas et al. compared the arterial phase, TNC, and VNC in 60 patients [21]. This study showed a significant difference in attenuation between TNC and VNC scans; however, it also showed a significantly higher attenuation of thyroid tissue compared to parathyroid adenoma in the VNC reconstruction. Leiva-Salinas et al. also showed similar diagnostic accuracy using a VNC and arterial images from a single dual energy CT acquisition compared to a biphasic protocol [21]. Maraia et al. showed a case report with two cases of VNC reconstruction using a dual-layer spectral CT scanner that was useful. However, our data showed that VNC not to be useful, because of the large variation in VNC values in our larger population. The difference is possibly due to a selection bias of cases in case reports, possible differences in reconstruction parameters between dual-layer scanners or algorithm versions and the larger variability in VNC in our larger cohort [19].

Our study had several limitations. First, this was a retrospective, single-institution study. Second, only one type of spectral CT technique was investigated, although this technique had not been investigated extensively in the literature. Most PAs showed enhancement type B and a small percentage showed enhancement type A, making subgroup analysis not possible. This is largely in line with the reported incidence of the enhancement patterns in the study by Bahl et al. However, the third type of enhancement (type C) was not present in our data, which limits generalizability [10]. A possible explanation could be the difference in scan protocol, as we used a post-threshold delay before scanning the contrast phases. PAs that showed type C enhancement in other studies were scanned without the use of post-threshold delay. In contrast, the study by Raeymaeckers et al. showed 89 % enhancement type A PAs (17/19) and only 11 % enhancement type B (2/19) [22]. Thirdly, the drawing of the ROI’s in a single slice could potentially introduce bias by not capturing heterogeneity within the lesions. Fourthly, variability could potentially be introduced by using different lymph nodes because the closest lymph nodes could be too small to analyze. Lastly, there was no assessment for inter- or intra-reader variability for ROI placement.

## 5 Conclusion

This study was the first to analyze the use of dual-layer spectral 4DCT reconstructions in a larger population. In this cohort, spectral reconstructions were found to be equally or less useful compared to conventional CT reconstructions in differentiating between thyroid, lymph node and PAs. In addition, VNC reconstructions are not recommended as a replacement for TNC because of the limited tissue differentiation until further research regarding more consistent VNC reconstructions has been done. Furthermore, research should be done to compare different types of spectral 4DCT for detecting PAs.

## CRediT authorship contribution statement

**Cornelis H. Slump:** Writing – review & editing, Supervision. **Marie Louise E. Bernsen:** Writing – review & editing, Supervision. **Frank B.M. Joosten:** Writing – review & editing, Methodology, Conceptualization. **Laura N. Deden:** Writing – review & editing, Methodology, Conceptualization. **Tessa Veerbeek:** Writing – review & editing, Writing – original draft, Methodology, Investigation, Formal analysis, Data curation, Conceptualization. **Krol Jorian P:** Writing – review & editing, Writing – original draft, Methodology, Investigation, Formal analysis, Data curation, Conceptualization. **Wim J.G. Oyen:** Writing – review & editing, Supervision, Methodology, Conceptualization.

## Ethics statement

This study was approved by the institutional research ethics board (Research number 2022-2154), which waived the requirement of written informed consent of each individual patient.

## Funding sources

This research did not receive any specific grant from funding agencies in the public, commercial, or not-for-profit sectors.

## Declaration of Competing Interest

The authors declare that they have no known competing financial interests or personal relationships that could have appeared to influence the work reported in this paper.

## Data Availability

The data that support the findings of this article are not publicly available due to patient privacy. They can be requested from the author at j.p.krol@utwente.nl.
